# NF-κB and Its Regulators During Pregnancy

**DOI:** 10.3389/fimmu.2021.679106

**Published:** 2021-05-05

**Authors:** Fernando Gómez-Chávez, Dolores Correa, Pilar Navarrete-Meneses, Juan Carlos Cancino-Diaz, Mario Eugenio Cancino-Diaz, Sandra Rodríguez-Martínez

**Affiliations:** ^1^ Secretaría de Salud, Cátedras CONACyT-Instituto Nacional de Pediatría, Mexico City, Mexico; ^2^ Secretaría de Salud, Laboratorio de Inmunología Experimental, Instituto Nacional de Pediatría, Mexico City, Mexico; ^3^ Departamento de Formación Básica Disciplinaria, Escuela Nacional de Medicina y Homeopatía-Instituto Politécnico Nacional, Mexico City, Mexico; ^4^ Dirección de Investigación, Universidad Anáhuac, Huixquilucan, Mexico; ^5^ Laboratorio de Genética y Cáncer, Instituto Nacional de Pediatría, Secretaría de Salud Mexico City, Mexico City, Mexico; ^6^ Laboratorio de Inmunomicrobiología, Departamento de Microbiología, Escuela Nacional de Ciencias Biológicas-Instituto Politécnico Nacional, Mexico City, Mexico; ^7^ Laboratorio de Inmunidad Innata, Departamento de Inmunología, ENCB-Instituto Politécnico Nacional, Mexico City, Mexico

**Keywords:** NF-κB, NF-κB regulation, IκBNS, IκBz, Bcl-3, pregnancy, reproductive system

## Abstract

The transcriptional factor NF-κB is a nuclear factor involved in both physiological and pathological processes. This factor can control the transcription of more than 400 genes, including cytokines, chemokines, and their modulators, immune and non-immune receptors, proteins involved in antigen presentation and cell adhesion, acute phase and stress response proteins, regulators of apoptosis, growth factors, other transcription factors and their regulators, as well as different enzymes; all these molecules control several biological processes. NF-κB is a tightly regulated molecule that has also been related to apoptosis, cell proliferation, inflammation, and the control of innate and adaptive immune responses during onset of labor, in which it has a crucial role; thus, early activation of this factor may have an adverse effect, by inducing premature termination of pregnancy, with bad outcomes for the mother and the fetus, including product loss. Reviews compiling the different activities of NF-κB have been reported. However, an update regarding NF-κB regulation during pregnancy is lacking. In this work, we aimed to describe the state of the art around NF-κB activity, its regulatory role in pregnancy, and the effect of its dysregulation due to invasion by pathogens like *Trichomonas vaginalis* and *Toxoplasma gondii* as examples.

## Introduction

According to global statistics, the leading cause of infant mortality and morbidity is premature labor (1). Worldwide, it is estimated that 1/10 of all pregnancies end preterm (2). According to the annual summary of vital statistics 2011-2012 of the American Academy of Pediatrics, 11.72% of all pregnancies in the United States end preterm, representing a 10.5% incidence increase from 1990 to 2012 (3). In developing countries, such as Mexico, the average preterm birth rate is similar to that in developed countries; however, this percentage can be as high as 40% in the poorest regions (4). Failure in identifying molecular mechanisms that limit and regulate the trigger of delivery has hampered its timely diagnosis, prevention, and treatment.

Labor is the last link between pregnancy and birth. It begins with the rupture of fetal membranes (chorion and amnion), followed by coordinated cervical dilation and uterine contractions, the fetus’s expulsion, and, finally, placental separation. The Nuclear Factor (NF-κB) partially induces this phenomenon, and thus, its activation before complete fetal development can cause preterm birth, increasing the risk for the mother, but especially for the fetus (5). Several studies have revealed the central role of NF-κB in labor regulation, controlling diverse pro-inflammatory cytokines that are upregulated in amniotic fluid, fetal membranes, placenta, myometrium, and cervix during normal and preterm labor (6–13). Thus, NF-κB is a cornerstone molecule that regulates the onset of labor induced by molecular stimuli such as cytokines, growth factors or hormones, but also by viral, bacterial, fungal, and parasite products (14–18).

## “Delivery” of NF-κB

NF-κB was first described by the group of Dr. T. David Baltimore as a component with nuclear activity (nuclear factor, “NF”) and DNA binding specificity, especially towards variations of the ten bp consensus DNA sequence of 5′-GGGRNYYYCC-3′ (in which R is a purine, Y is a pyrimidine, and N is any nucleotide), known as κB sites (19). It was initially demonstrated that NF-κB induced gene expression of the immunoglobulin kappa light-chain in antibody-producing B cells (thus the “kB” component of its name) (20). Later, it was clear that this was a family of proteins composed by two members, the NF-κB and the Rel subfamilies associated with several biological phenomena, such as immunity and development, as well as diseases like cancer and inflammatory disorders (17). These molecules are also related to pregnancy phenomena, including normal and preterm delivery (5, 21).

## NF-κB Family

**Figure 1:**
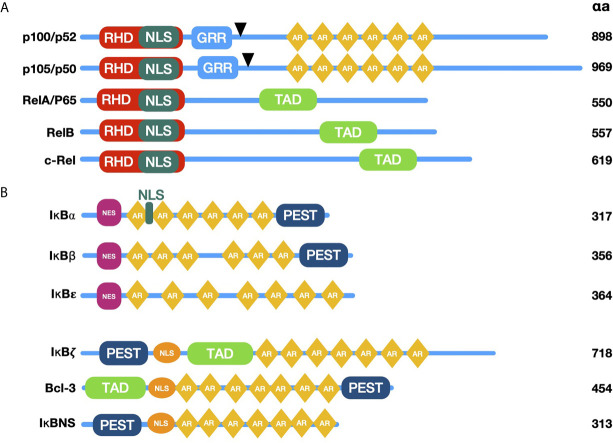
Schematic diagram of NF-κB and IκB family members. **(A)** The proteins p100 and p105 are the precursors of p52 and p50; they lack transactivation domain (TAD), which bind to other proteins such as transcription factors coregulators. Black arrowheads are pointing to C terminal proteolytic cleavage sites originating p52 and p50. NF-κB family proteins contain sequences required for DNA binding, dimerization, and nuclear localization, called Rel Homology Domain (RHD). **(B)** Typical and Atypical members of the NF-κB inhibitors (IκB) family are characterized by the presence of Ankyrin Repeats (AR) and their ability to bind and sequester NF-κB dimers in the cytoplasm (typical members) or recruit them to specific gene promoters in the nucleus (atypical members). RHD, Rel homology domain; TAD, transactivation domain; GRR, glycine-rich region; AR, ankyrin repeats; PEST, proline-, glutamic acid-, serine threonine-rich sequence; NLS, Nuclear Localization Signal; NES, Nuclear Export Signal.

The classic NF-κB family is composed by five members: NF-κB1 (p105/p50), NF-κB2 (p100/p52), p65 (RelA), c-rel and RelB (18) (**Figure 1A**). These proteins share a conserved N-terminal region designated as Rel homology domain (RHD), which mediates dimerization, binding to DNA, translocation to the nucleus, and interaction with NF-κB inhibitory proteins (IκB’s) (22, 23) (**Figure 1B**). The active forms of NF-κB are homo- or heterodimers of various family members (18). Twelve to 15 possible dimers formed by the NF-κB members’ interaction can bind DNA and, therefore, can regulate gene transcription (18). The diversity of combinations formed by NF-κB members contributes to the specificity of several panels of regulated genes (18, 24, 25). Due to this specificity for DNA binding sites, dimers have different protein-protein interactions with target promoters and are activated under particular physiological conditions (25).

Proteins p65, c-rel, and RelB contain transcriptional activation domains (TADs) in their C-termini, required for NF-κB dimer translocation to the nucleus (24). In contrast, family members p50 and p52 lack TADs, but can form heterodimers with TAD carrying proteins, modifying the specificity for κB sites; repressing transcription by blocking κB binding sites in homodimers or promoting recruitment to other proteins containing TADs (26). Proteins p50 and p52 are generated by the proteolytic processing of their respective precursors, p105 and p100 (27). The latter are constitutively processed, although p105 is more efficiently cut. Most cells exhibit high levels of p50, whereas the levels of p52 are steadily lower (28, 29). Despite the diversity of NF-κB dimer combinations, the most prevalent NF-κB heterodimer is formed by p50 and p65, which is typically bound to one of its inhibitors in the cytoplasm of non-stimulated cells (30).

## NF-κB Regulators

**Figure 2:**
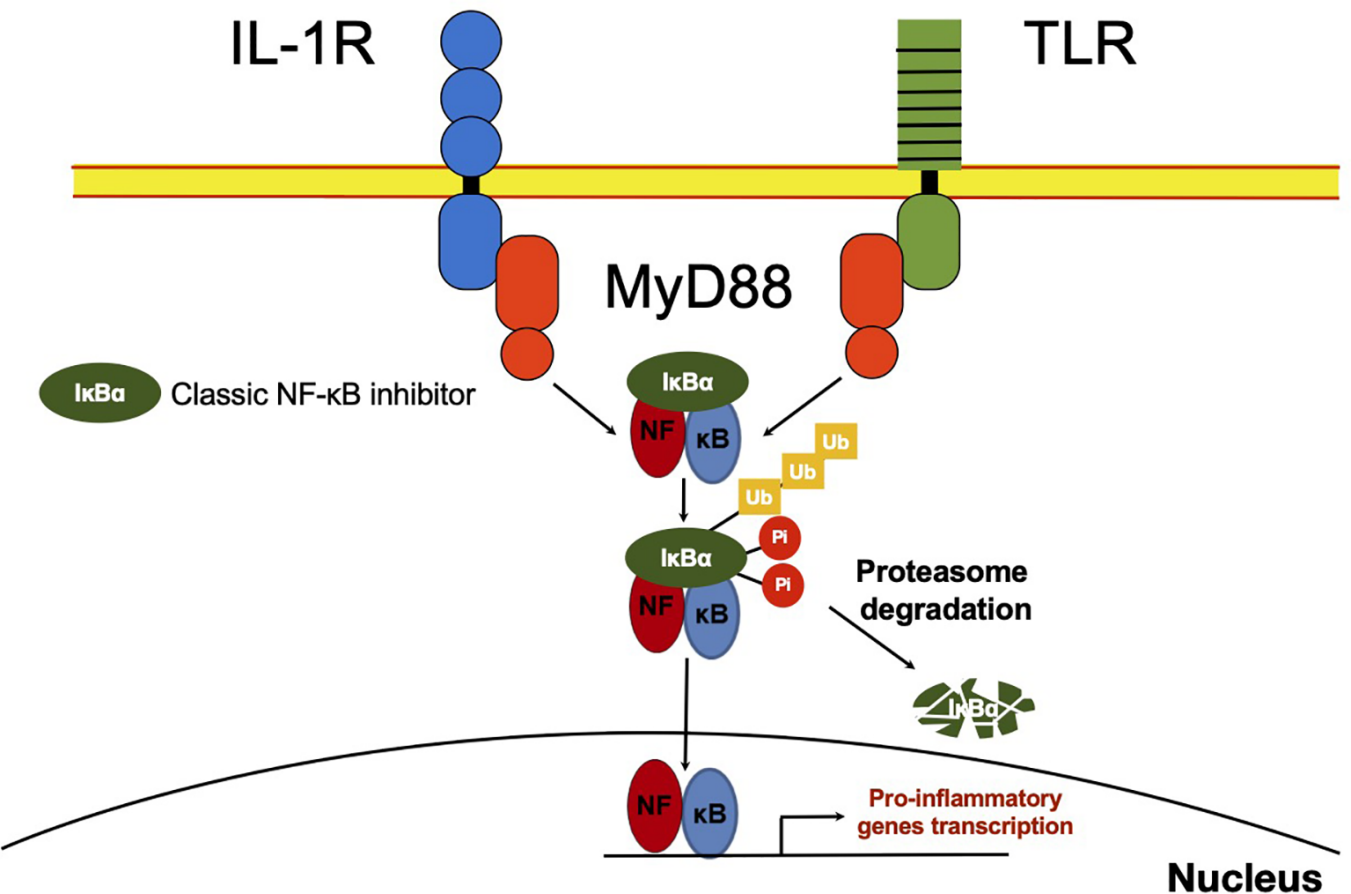
NF-κB classic activation. After recognition of IL-1R or TLR ligands, MYD88 promotes activation of the IKK complex, which mediates IκB phosphorylation. IκB⍺ phosphorylated, in turn, is ubiquitinated and delivered to the proteasome where it is degraded. NF-κB released in the cytoplasm can now translocate to the nucleus and promote transcription of several primary response genes. Most of these genes are related to inflammation such as TNFα, IL-1β, IκBζ and IκBNS.

As soon as it was described, it was inferred the capability of NF-κB to interact with other molecules in the cytoplasm. The presence of NF-κB was demonstrated in the cytoplasm of non-stimulated cells by treating cell cytoplasmic fractions with dissociating agents, such as the weak detergent sodium deoxycholate (31). This observation suggested a non-covalent interaction with an inhibitory molecule responsible for maintaining NF-κB in an inactive state (31, 32). Later, these molecules capable to bind NF-κB in the cytoplasm [NF-κB inhibitor alpha (IκBα), NF-κB inhibitor beta (IκBβ) and NF-κB inhibitor epsilon (IκBϵ)] were described and considered as classical regulators of NF-κB activity (33–35) (**Figures 1B** and **2**). The IκBs characteristically present ankyrin repeat domains, which interact with the RHD in NF-κB, limiting nuclear localization and DNA binding (33, 34, 36). The precursor proteins p105 and p100 also contain ankyrin repeats, and they function as IκB proteins (37–39). These considered non-classical IκBs, are importantly involved in determining the formation of new dimers of NF-κB *via* the processing and assembly of large complexes with IκB activity (39). Crystallographic and mechanistic studies have revealed that IκBs, specifically IκBα, acts on the dimer p50/p65, masking the nuclear localization sequence (NLS) of the p65 subunit. Although p50 NLS is still exposed in the IκBα/p50/p65 trimer, the presence of the nuclear export sequence (NES) present in IκBα and p65, results in an active nucleus to cytoplasm, and cytoplasm to nucleus shuttle of this complex (40). Because the export process is more efficient than the import process, cellular localization of IκBα bound to NF-κB is preferably in the cytoplasm of non-stimulated cells (40). IκBβ lacks NES and masks both NF-κB (p50/p65) NLS, and thus, this complex remains sequestered in the cytoplasm of non-stimulated cells (41).

**Figure 3:**
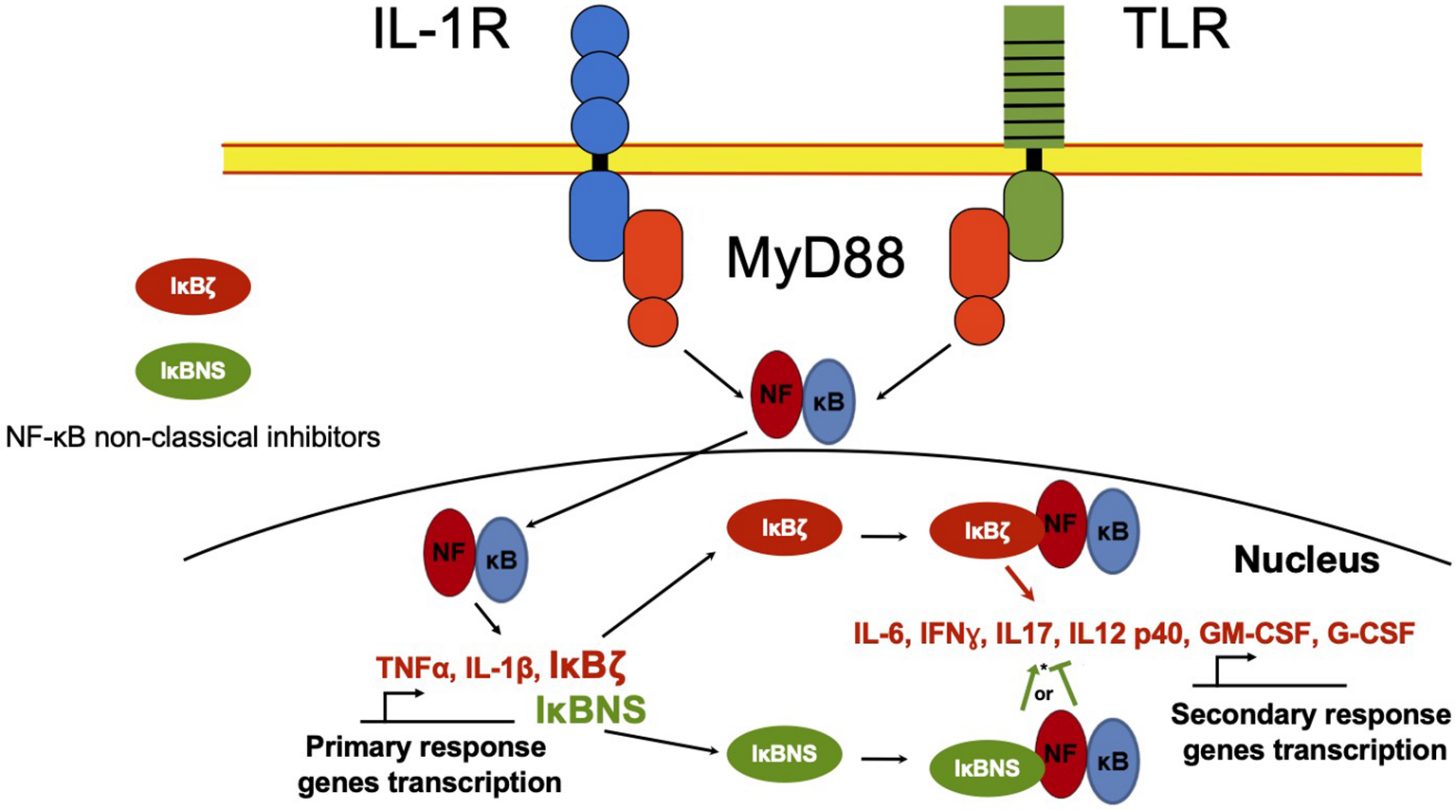
NF-κB non-classical regulators in the nucleus. NF-κB in the nucleus regulates the transcription activity of primary response genes. Non-classical NF-κB regulators such as IκBζ and IκBNS proteins, can interact with NF-κB and recruit it to the promoter of secondary response genes like IL-6 or IFNγ, inducing or inhibiting gene transcription. *IκBNS can act as NF-κB promoter or inhibitor depending on cell type and environment.

Besides the classical proteins IκB, other non-classical IκB proteins share ankyrin repeats (**Figure 1B**). Unlike classical IκBs, these are not generally expressed in unstimulated cells, but are preferably induced after cell activation by several stimuli, like IL-1β or TLR ligands in a spatiotemporal fashion, controlling gene transcription of secondary response genes (**Figure 3**) (42).

## Bcl-3

Bcl-3 was the first cloned protein belonging to the family of non-classical IkBs. Bcl-3 was initially identified as an oncogene present in chronic lymphocytic leukemia (43). Bcl-3 was described as an inhibitor of the NF-κB activity, specifically bound to heterodimers containing the p50 subunit (44, 45). Subsequent studies revealed that Bcl-3 could also act as a transcriptional coactivator of p50 homodimers (46). It has been described that Bcl-3 is able to bind p50 and p52 homodimers, which lack TADs. In contrast to p50 and p52, Bcl-3 poses a distinctive TAD region. The binding of Bcl-3 to p50 or p52 provides the complex with transcriptional activity (47, 48). Binding of Bcl-3-p52-p52 or Bcl-3-p50-p50 complexes to their respective promoters can control the expression of the cyclin D1 and the epidermal growth factor receptor (EGFR) (49). A large study reported Bcl-3-p50-homodimer-dependent genes associated with disuse muscle atrophy; such genes are *Trim63* (*MuRF1*), *Fbxo32 (MAFbx)*, *Ubc, Ctsl*, *Runx1*, *Tnfrsf12a* (Tweak receptor) and *Cxcl10* (*IP-10*) (50). In contrast, Bcl-3 also stabilizes homodimers of NF-κB bound to DNA, repressing its transcriptional activity (51). In this context, Bcl-3 has been involved in processes of tolerance to LPS (51). It is known that treatment of immune cells with IL-10 decreases the DNA binding of NF-κB and induces Bcl-3 expression (52). The DNA binding activity of NF-κB and the consequent production of TNF-α are diminished in macrophages of the colonic *lamina propria* -significant IL-10 producers- stimulated with LPS. Moreover, Bcl-3 deficient macrophages show defects in suppressing the production of TNF-α but not IL-6, which is a cytokine also regulated by NF-kB. This suggests that Bcl-3 is involved in the suppression of the innate immune response by regulating the expression of specific genes such as TNF-α (52). Regarding pregnancy, Bcl-3 is overexpressed in human placentas of severe early-onset preeclampsia cases (53). Recently, Bcl-3 has been reported in normal uterus of mice, where its subtle expression correlates with low production of TNF-α (54).

## IκBζ

The IκBζ protein is another non-classical IκB, which exhibits greater homology to Bcl-3 than to other IκBs. The first report of this molecule was presented in a paper that sought to identify upregulated genes upon cells challenged with LPS (55). Unlike the classical IκBs, IκBζ is neither constitutively expressed nor controlled by inducible degradation, but post-transcriptionally regulated by microRNA (miR)-124a (56, 57). Kitamura et al. found that IκBζ was positioned in the nucleus upon the challenge with LPS, where it stimulated the production of IL-6 (55). Soon later, it was determined that IκBζ was also induced by IL-1 but not by TNF-α in mice, and similar to the former study, it was localized in the nucleus (58). In contrast to the first reports, it was shown that IκBζ could be considered a new negative NF-κB regulator, acting in the nucleus by association either the p50 or the p65 subunit (56, 58, 59). IκBζ has been linked to the production of IL-6 in response to the challenge with TLR ligands, preferentially binding to p50 NF-κB dimers (60–63). IκBζ has also been involved in the production of IFN-γ (64), CCL2/MCP-1 (65), neutrophil gelatinase-associated lipocalin (NGAL) (66), human β -defensin 2 (66), antibacterial protein lipocalin-2 (Lcn2) (67), and IL-17A during Th17 polarization (68). Congruently, IκBζ downregulates Foxp3 in T cells, IL-10, CTLA-4, and the class switch DNA recombinase activation-induced cytidine deaminase (AID) in B cells, as well as IL-12 and IL-18 in activated -mouse and human- NK cells (62, 69–72).

Interestingly, it has been reported that the 10-hydroxy-trans-2-decenoic acid (10H2DA), a major fatty acid component of royal jelly, presents an inhibitory effect on LPS-induced IL-6 production by downregulating IκBζ expression in RAW 264 murine cell line. Although the pathway by which IκBζ is downregulated is unknown, 10H2DA showed to be also an important expression modulator of second response genes regulated by IκBζ, such as Lipocalin, G-CSF, and IL-6, but not TNF-α (73). Specifically in pregnancy, IκBζ is overexpressed in human myometrium in spontaneous human labor at term (16). Interestingly, magnesium sulfate (MgSO4), given to woman at risk of preterm labor, provides fetal neuroprotection, which can be explained by its ability to inhibit inflammation during pregnancy and particularly to reduce the expression of pro-inflammatory cytokines and their transcription regulator IκBζ, as seen in both human placental explants and a rat model of pregnancy (74). More recently, our group has reported how Galectin-1, a lectin able to bind β-galactosides added to other proteins by glycosylation, reduces the expression and production of IL-6 in human decidua cells challenged with LPS *in vitro*, through downregulation of IκBζ expression, its translocation to the nucleus, and its recruitment to the IL-6 promoter (75).

## IκBNS

IκBNS was initially defined as a rapidly induced gene upon thymocyte TCR stimulation, which inhibited NF-κB DNA binding activity, but not its translocation to the nucleus, suggesting that it can negatively regulate NF-κB within the nucleus (76).

Later, the expression of IκBNS was identified in macrophages of the *lamina propria* in the colon, while it was undetectable in peripheral blood monocytes. IκBNS was shown selectively recruited to the IL-6 but not the TNF-α promoter, suppressing LPS-induced IL-6 production (77). IκBNS in macrophages and DCs was demonstrated to be a regulator for IL-6 and IL-12p40 transcription, cytokines induced by several TLR ligands, supporting the idea that IκBNS is a negative NF-κB regulator (77, 78). In apparent contradiction, IκBNS KO mice present a reduced proliferation of T cells, which was associated with IκBNS positive control of IL-2 expression through its gene promoter binding. These results suggest that this non-classic IκB might be differentially involved in positive and negative regulation of cytokine expression, depending on the cell type and the environmental conditions (77–79). IκBNS has been related to innate-like, early B and plasma cell functions, since IκBNS KO mice lack B1 cells and impaired marginal B cell zones development (80). In this context, Arnold et al. reported that IκBNS is required for extrafollicular responses to T-independent and T-dependent immunogens, as well as natural IgM antibodies production (81–83). More recently, B cell impaired development in IκBNS KO mice was related to the role of IκBNS as an enhancer of follicular helper T cells differentiation and function because IκBNS is essential for the induction of Bcl-6 and IL-21 (84). IκBNS, like Bcl-3, can be induced after stimulation with LPS in regulatory dendritic cells (rDC) and in a B-10 cell subpopulation, which induces the production of high levels of IL-10 (85, 86).

Interestingly, IκBNS can drive Foxp3 expression *via* association with the Foxp3 gene promoter, stimulating Treg cell development in the thymus during gut inflammation *in vivo* (87). IκBNS has also been involved in generating Th-17 cells in experimental autoimmune encephalomyelitis (EAE) (88, 89). All this contradictory evidence indicates that IκBNS function depends on cell type and microenvironment, which determine its role as a positive or negative regulator.

Although poorly investigated in reproduction, IκBNS is an interesting molecule. Our group found it is expressed in pro-estrus, and poorly synthesized during estrus (while IL-6 is over-produced), an inflammatory phase in the estrous cycle of mice. In contrast, IκBNS is overexpressed while IL-6 is downregulated in metestrus, a cycle phase characterized by the development of the *corpus luteum*, increased progesterone secretion, and decreased estrogen secretion (54).

More recently, we have also reported that in the uterine tissue of pregnant mice, the regulatory effect of IκBNS over IL-6 is evident in an *L. monocytogenes* infection model: IL-6 overexpression was promoted by low expression of IκBNS, which provoked fetal growth restriction and resorption (90).

## Activation of NF-κB by the IKKs

IKKα, IKKβ and IKKy (also called NEMO, NF-κB essential modulator) compose the IKK complex that phosphorylates Ser and Thr residues of NF-κB inhibitors, such as IκBα, labeling them for their ubiquitination and degradation by the proteasome, allowing in this way the release of NF-κB for its translocation to the nucleus (reviewed by Echeverria et al. (91)). Despite the role of other kinases, IKKs are especially important since they act rapidly, promoting classical IκBs degradation. The various members of this complex are also under the control of molecules responsive to PAMPs or DAMPs; for example, NEMO degradation is promoted by the E3 ubiquitin ligase TRIM29, which in this way maintains immunological homeostasis after infection, for example by influenza virus (92).

## NF-κB in Pregnancy: Expression in Fetal and Maternal Tissues

As previously discussed, activation of NF-κB implies that its dimers are released into the cytoplasm and can thus translocate to the nucleus and bind DNA *via* the κB motifs of NF-κB-regulated gene promoters. These κB motifs have been identified in several genes of pro-inflammatory mediators, such as adhesion molecules (ICAM-1); enzymes like inducible NO synthase, phospholipase A2S, cyclooxygenase-2 (COX-2) and metalloproteinases (MMP-9); cytokines (IL-1β, IL-6, TNF-α); and chemokines such as IL-8. These genes are widely expressed during normal pregnancy and in some gestational disorders (93–95).

The activity of NF-κB has been indirectly observed by the increase of pro-inflammatory cytokines such as IL-1β, TNF-α, IL-6, IL-8, or IFN*γ* in the amniotic fluid, the placenta, the fetal membranes, the myometrium, the decidua and the cervix (96–101). Immune cells that infiltrate the fetal-mother interface can secrete chemokines and cytokines, leading to activation of NF-κB in the myometrium, the cervical epithelium, and the amnion cells (99). Many components of the signaling pathway of the NF-κB have been identified in pregnancy tissues. It has been shown that in the first-trimester decidua, there is the expression of IκBɑ, IKK complex, and NIK (102). On the other hand, the expression of genes regulated by NF-κB is increased by the end of pregnancy, as well as its DNA binding activity in isolated cytotrophoblasts, in primary cultures of the amnion, and in nuclear extracts prepared from the placenta, the amnion, and the choriodecidua (9, 103–106).

## NF-κB Regulation During Pregnancy

Substantial evidence supports the notion that pregnancy is significantly regulated by cytokines and hormones, driving different pathways that lead to the activation of specific nuclear factors, including NF-kB, which controls the expression of several molecules that can promote labor under normal conditions or can induce preterm birth caused by infectious and non-infectious disorders of pregnancy (93). Before gestation, NF-κB activity is present in the female genital tract and has an essential role in regulating innate immune response, because a suboptimal response could favor a permissive environment for pathogen colonization, whereas an over-induced response could cause excessive inflammation and tissue damage (54, 107). Cytokines produced under NF-κB regulation play a critical role in human implantation, inducing adhesion molecules’ expression on the embryo and the maternal surfaces, regulating by these means the expression of proteases that remodel the extra-cellular matrix, and promoting the invasion and differentiation of trophoblasts (108, 109). Once implantation has occurred, excessive activation of NF-κB can activate an uncontrolled response, potentially contributing to disorders of fetus development, such as intrauterine growth restriction, abortion, or preterm birth (10, 103, 110, 111). During pregnancy, NF-κB is negatively regulated in the maternal peripheral blood T cells (112, 113). Also, hormones like progesterone (P4), importantly elevated during pregnancy, can suppress the activity of NF-κB (114). Likewise, cytokines such as IL-10 have a vital role in downregulating NF-κB at the maternal-fetal interface and systemically (115, 116). IL-10 is expressed during the most extended period of pregnancy, both in humans and mice, and different studies have demonstrated its ability to downregulate TNF-ɑ, IL-6, and prostaglandins in human fetal membranes and decidual cells (115–117). Moreover, the relationship between NF-κB activity, pro-inflammatory cytokines, and preterm birth was demonstrated in IL-10 KO mice: the absence of this cytokine resulted in an increased expression of IL-6 and TNF-ɑ induced by LPS, which caused the onset of early labor (118).

More recently, the important role of Galectins has been introduced in reproductive biology, e.g., Gal-1 is abundantly produced in the maternal reproductive tissues in humans and mouse, suggesting a crucial role in the development of maternal tolerance to the fetus during pregnancy, by inhibition of TNF-ɑ and IL-6 expression, induction of IL-10 and promotion of regulatory T cells (Treg) proliferation (119–121). Interestingly, it has been demonstrated that Gal-1 regulates pro-inflammatory cytokine production by blocking NF-κB activation in peripheral blood monocytes (122). Besides, our group has shown that Gal-1 reduces the effect of LPS on IL-6 production in non-immune cells from the fetal-maternal interface, such as decidual fibroblasts; even more, we found that Gal-1 inhibits the nuclear translocation ability of IkBz and its recruitment to the IL-6 promoter in LPS treated cells (75).

Regulation of NF-κB is crucial during pregnancy, and thus, it is not surprising this transcription factor has already been proposed as a key target for preterm labor prevention (123). Even more, *in sillico* models have been used to analyze the inhibitory effect over NF-kB, simulating an anti-inflammatory treatment to avoid the development of preterm labor, which remarks the therapeutic implications of NF-κB downregulation (124). Although the exact mechanism of NF-κB regulation during pregnancy has not been established, it is clear that its activity should remain suppressed during most normal gestation time until the end.

## NF-κB Dysregulation by Pathogens in Pregnancy: Examples of Two Protozoa

During pregnancy, the mother’s immune system is highly regulated but can effectively respond against pathogens. Nevertheless, best-adapted pathogens have evolved so they modulate NF-κB activation, limiting the response against them and favoring the pathogen survival (125). *Trichomonas vaginalis* is a genitourinary tract lumen-dwelling flagellated protozoan that infects humans (126). It is responsible for trichomoniasis, one of the most common non-viral sexually transmitted diseases (127). Infection by *T. vaginalis* has been associated with adverse outcomes of pregnancy like low birth weight and preterm labor. It has been suggested that these adverse outcomes are mediated by downregulation of TNF-α and IL-12 expression through blocking NF-κB translocation to the nucleus (128, 129), but it induces IL-1β production in human prostate epithelium through activation of ROS (130), and through this mechanism it also provokes apoptosis of the host cells, including monocytes and primary cultures of human vaginal epithelial cells by means of NF-κB downregulation (131–134).

Another pathogen related to pregnancy is *Toxoplasma gondii*, the causal agent of toxoplasmosis, a cosmopolitan, water/foodborne infection that can be transmitted to the fetus which may cause severe pathological conditions (135, 136). This protozoan is a strict intracellular pathogen, classified in three classical strains and the atypical variants, some of them with the capacity to modulate NF-κB (137). For invasion, *T. gondii* secretes proteins from the parasite organelles called rhoptries (ROP) and dense granules (GRAs) into the cytoplasm of the host cell, which modulate molecular host signaling and transcription (138). ROP and GRA proteins have been involved in the control of NF-κB activation. The GRA15 protein of type II strains activates NF-κB, leading to a pro-inflammatory environment, which results in disease manifestations like encephalitis and colitis. Type II *T. gondii* strains are more prevalent in human congenital toxoplasmosis; interestingly, these strains allow the recruitment of immune cells that can be infected by the parasite and are useful to disseminate it throughout the fetus body (139–142). On the other hand, ROP 18 from *T. gondii* strain I (GRA15-type I cannot induce NF-kB) can directly interact with NF-κB p65 and phosphorylate it at Ser-468, targeting p65 for proteasome degradation, this manipulation of the host immune system facilitates infection (143). Importantly, there is a low number of reported congenital toxoplasmosis cases due to type I strains, but these cases commonly present pregnancy complications, including abortion (141). Congenitally infected patients may develop pathological conditions such as hydrocephalus, macro or microcephalus, cerebral calcifications, retinochoroiditis, and other ocular or central nervous system alterations, which can manifest even years later in life and most severe congenital infection cases can cause spontaneous abortion or stillbirth (144). We have recently shown evidence that *T. gondii* congenital transmission and severity of clinical manifestations in the infected newborns depend on the promotion of an inflammatory non-regulated environment, in which NF-κB and its regulators are probably involved (145, 146).

## Conclusions

**Figure 4:**
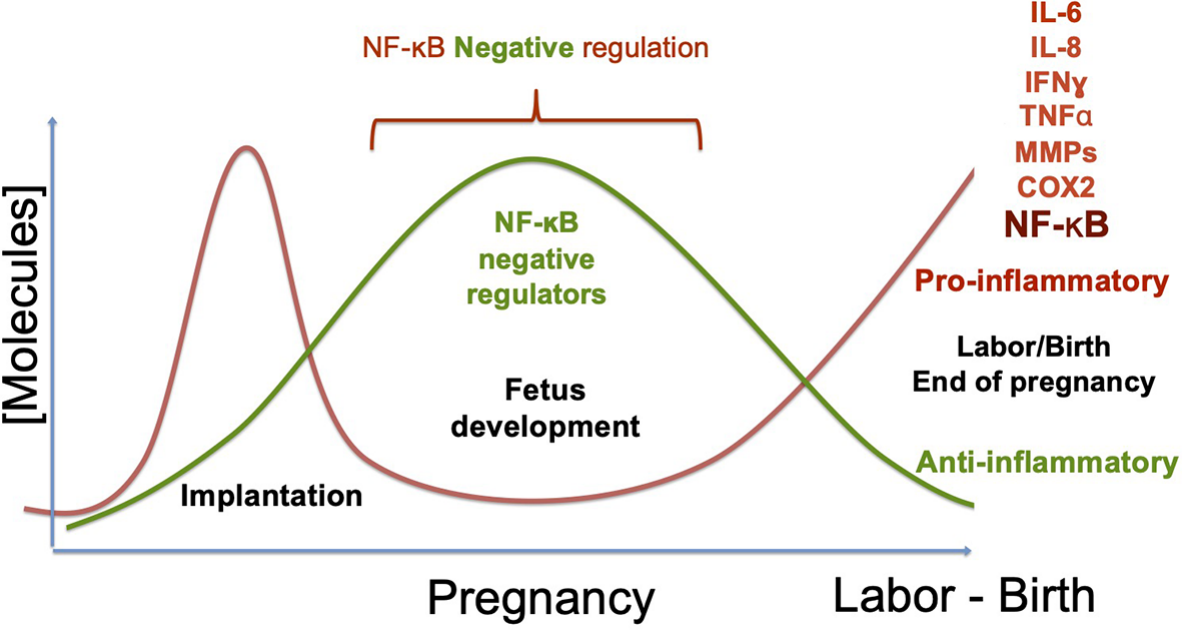
NF-κB regulation during pregnancy. During pregnancy, two critical changes in the profile of molecules produced at the maternal-fetal interface and the systemic level must occur. Implantation requires developing an inflammatory phenotype, which depends on the NF-κB´s activity. In uncomplicated pregnancies, the pro-inflammatory environment has to change towards an anti-inflammatory phenotype during the fetus’s development, downregulating NF-κB. Once the fetus’s development is complete, anti-inflammatory molecules’ production decreases, and inflammatory molecules’ expression is triggered again. This breaking point where the phenotype changes from anti-inflammatory to inflammatory initiates labor, inducing molecules such as IL-6, IL-8, TNF-ɑ, Metalloproteinases, and COX2, now regular contractions and rupture of the fetal membranes begin. As a consequence of the change to an inflammatory environment, the new individual’s birth is promoted. The primary complications of pregnancy occur when labor begins prematurely; that is, when the profile change occurs before the fetus complete development, which promotes pre-term pregnancies or even abortion. Therefore, down-regulation of inflammatory molecules is necessary during fetal development, remarking the prominent role of NF-κB activity regulation in the required inflammatory/anti-inflammatory balance.

**Figure 5:**
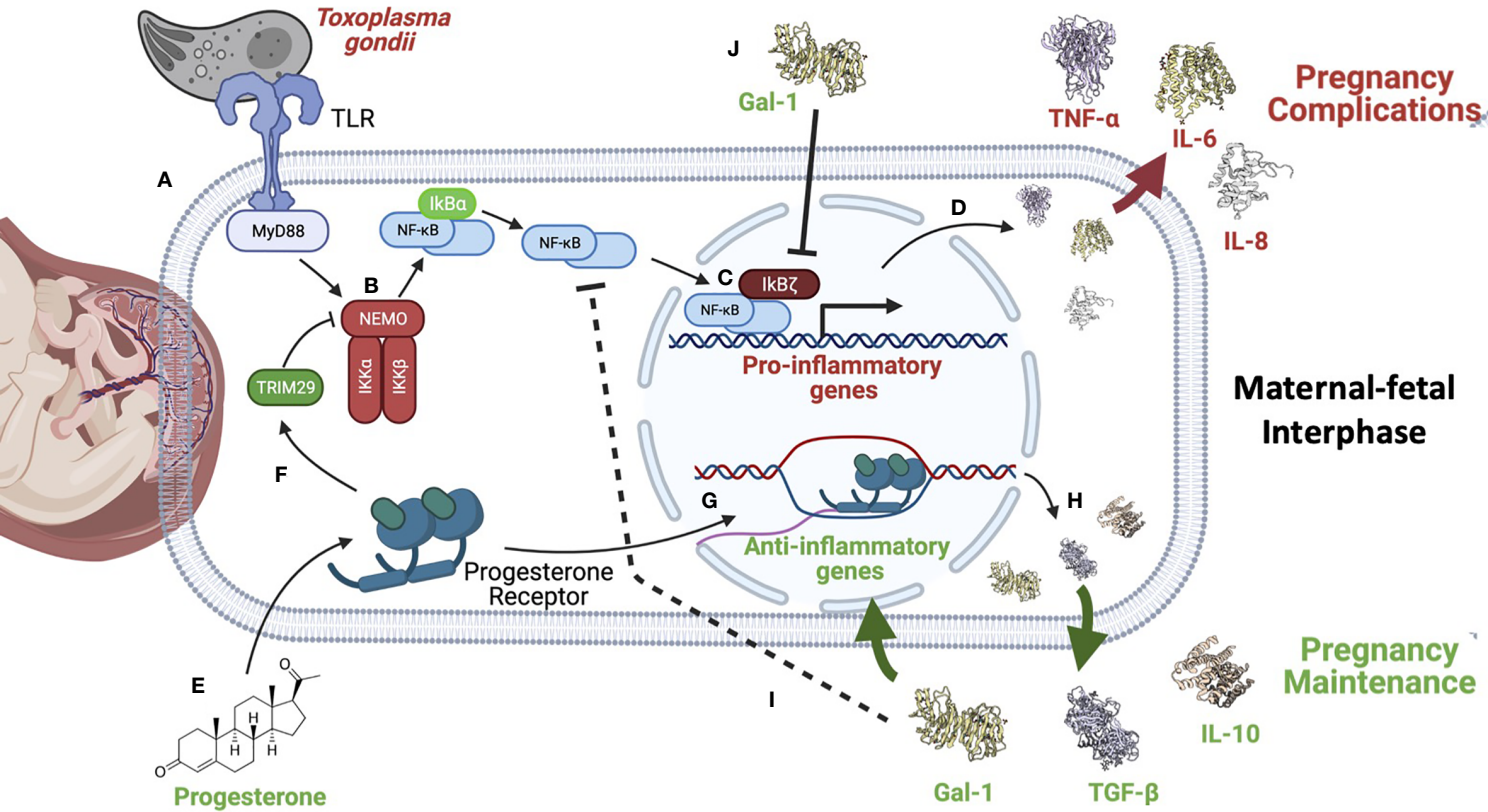
NF-κB is a master regulator of pregnancy development. NF-κB activation can be caused by different stimuli such as pathogens like the zoonotic parasite *Toxoplasma gondii* infection during pregnancy. **(A)** This parasite is recognized by TLR-2 and TLR-4, triggering the activation of MYD88, which results in **(B)** the activation of the IKK complex, inducing IκBα phosphorylation and its degradation by the proteasome. Now, NF-κB free in the cytoplasm **(C)** can translocate itself to the nucleus where, as discussed earlier, it can be helped by its non-classical regulators, such as IkBζ **(D)** to promote the expression of pro-inflammatory cytokines, like IL-6. Overproduction of pro-inflammatory mediators can favor *T. gondii* vertical transmission to the fetus, and worsen the severity of clinical features such as intrauterine growth restriction, pre-term, or even abortion (145, 146). **(E)** On the other hand, several reports have shown that anti-inflammatory molecules highly produced during pregnancy, like progesterone, can down-regulate NF-κB **(F)**; this hormone can induce TRIM29 (149), which promotes NEMO degradation, inactivating the IKK complex, and in this way turning off the NF-κB pathway. **(G)** Progesterone can also promote the expression of other anti-inflammatory genes **(H)** perpetuating an anti-inflammatory environment required for pregnancy maintenance. **(I)** Gal-1, TGF-β, and IL-10 have been widely described as potent NF-κB inhibitors during pregnancy. **(J)** Gal-1 in cells from the maternal-fetal interphase can limit IkBζ translocation to the nucleus, inhibiting NF-κB recruitment to the promoters of pro-inflammatory cytokines, such as IL-6 (75). Created with BioRender.com.

Labor can be promoted normally, or by exposure to damaging substances, infectious agents, or genetic predisposition (12, 13, 147, 148). In all cases, it depends on the development of an inflammatory flux orchestrated by the transcription factor NF-kB. Its activation begins the cascade of events that culminate with the onset of labor and the rupture of the fetal membranes, which depends on the production of pro-inflammatory cytokines, chemokines, metalloproteinases, and prostaglandin-synthesis enzymes, among other molecules. In a normal pregnancy, this final step elicits the birth of a new individual (**Figure 4**). In contrast, NF-κB activation before the full development of the fetus can induce product disease or loss, as well as and collateral damage to the mother. Some insights on the mechanisms of NF-κB activation/regulation during pregnancy complications are shown in **Figure 5**, in which the balance of pro-inflammatory stimuli and anti-inflammatory environment determines the success or failure of the pregnancy. Although there is some information about NF-κB activity at the beginning and the end of pregnancy, there is very little information on the dynamics of its functions and regulation along the most extended period of pregnancy, so it is expected it will be studied furthermore in the near future.

## Author Contributions

All authors contributed to the article and approved the submitted version.

## Funding

This work was supported by the Instituto Nacional de Pediatría (040/2018) and by SIP-IPN (SIP20210419, SIP20210140, and SIP20210139). FG-C is a Cátedra CONACyT Research Fellow. All authors are SNI-CONACyT fellows.

## Conflict of Interest

The authors declare that the research was conducted in the absence of any commercial or financial relationships that could be construed as a potential conflict of interest.
